# Algorithmic extraction of smartphone accelerometer-derived mechano-biological descriptors of resistance exercise is robust to changes in intensity and velocity

**DOI:** 10.1371/journal.pone.0254164

**Published:** 2021-07-20

**Authors:** Claudio Viecelli, David Aguayo, Samuel Dällenbach, David Graf, Basil Achermann, Ernst Hafen, Rudolf M. Füchslin

**Affiliations:** 1 Institute of Molecular Systems Biology, ETH Zurich, Zurich, Zurich, Switzerland; 2 Kieser Training AG, Zürich, Zurich, Switzerland; 3 Institute of Applied Mathematics and Physics, Zurich University of Applied Sciences, Zurich, Winterthur, Switzerland; 4 ieffects AG, Zurich, Switzerland; 5 European Centre for Living Technology, Ca’ Bottacin, Venice, Italy; Baylor College of Medicine, UNITED STATES

## Abstract

**Background:**

It was shown that single repetition, contraction-phase specific and total time-under-tension (TUT) can be extracted reliably and validly from smartphone accelerometer-derived data of resistance exercise machines using user-determined resistance exercise velocities at 60% one repetition maximum (1-RM). However, it remained unclear how robust the extraction of these mechano-biological descriptors is over a wide range of movement velocities (slow- *versus* fast-movement velocity) and intensities (30% 1-RM *versus* 80% 1-RM) that reflect the interindividual variability during resistance exercise.

**Objective:**

In this work, we examined whether the manipulation of velocity or intensity would disrupt an algorithmic extraction of single repetitions, contraction-phase specific and total TUT.

**Methods:**

Twenty-seven participants performed four sets of three repetitions of their 30% and 80% 1-RM with velocities of 1 s, 2 s, 6 s and 8 s per repetition, respectively. An algorithm extracted the number of repetitions, single repetition, contraction-phase specific and total TUT. All exercises were video-recorded. The video recordings served as the gold standard to which algorithmically-derived TUT was compared. The agreement between the methods was examined using Limits of Agreement (LoA). The Pearson correlation coefficients were used to calculate the association, and the intraclass correlation coefficient (ICC 2.1) examined the interrater reliability.

**Results:**

The calculated error rate for the algorithmic detection of the number of single repetitions derived from two smartphones accelerometers was 1.9%. The comparison between algorithmically-derived, contraction-phase specific TUT against video, revealed a high degree of correlation (r > 0.94) for both exercise machines. The agreement between the two methods was high on both exercise machines, intensities and velocities and was as follows:

LoA ranged from -0.21 to 0.22 seconds for single repetition TUT (2.57% of mean TUT), from -0.24 to 0.22 seconds for concentric contraction TUT (6.25% of mean TUT), from -0.22 to 0.24 seconds for eccentric contraction TUT (5.52% of mean TUT) and from -1.97 to 1.00 seconds for total TUT (5.13% of mean TUT). Interrater reliability for single repetition, contraction-phase specific TUT was high (ICC > 0.99).

**Conclusion:**

Neither intensity nor velocity disrupts the proposed algorithmic data extraction approach. Therefore, smartphone accelerometers can be used to extract scientific mechano-biological descriptors of dynamic resistance exercise with intensities ranging from 30% to 80% of the 1-RM with velocities ranging from 1 s to 8 s per repetition, respectively, thus making this simple method a reliable tool for resistance exercise mechano-biological descriptors extraction.

## Introduction

Adaptations to resistance exercise are highly specific [1] and depend on mechano-biological descriptors such as muscle action [2, 3], movement velocity [4–6], range of motion [7], muscle groups [8], involved energy systems [9], intensity and training volume [10].

Movement velocity, for example, has been shown to increase cross-sectional area (CSA) and muscle thickness of knee extensors [11, 12] and CSA of quadriceps [13] and biceps brachial [14] to a greater extend when comparing slow *versus* fast movement velocities. Movement velocities stimulate muscle protein synthesis rate responses differently [6], which substantially impacts hypertrophy. Given the importance of these variables, movement velocity is often overlooked [15] or neglected [16].

Resistance exercise consists of mechano-biological descriptors, comprising load magnitude, number of repetitions, number of sets, rest in-between repetitions ([s] or [min]), number of exercise interventions (per [d] or week), duration of the experimental period ([d] or weeks), fractional and temporal distribution [s] of one repetition, rest in-between repetitions([s] or [min]), time under tension ([s] or [min]), volitional muscular failure, range of motion, recovery time in-between exercise sessions ([h] or [d]) and anatomical definition of exercise (exercise form) (for an extensive review see [16]).

Up to date, still only classical resistance exercise mechano-biological descriptors such as intensity (*e*.*g*. in % 1-repetition maximum [RM] or [kg]), number of repetitions, number of sets, rest in-between sets (*e*.*g*. in [s] or [min]), number of exercise interventions (*e*.*g*. per [d] or week) or duration of the experimental period (*e*.*g*. in [d] or weeks) are reported. As seen above, contraction-specific phases per repetition (*e*.*g*. in [s]), for example, is important to interpret different muscular adaptations to otherwise seemingly identical training protocols.

Just recently, Viecelli *et al*. [17] proposed an approach using smartphone accelerometer data from a machine-based dynamic resistance exercise to extract contraction-phase specific temporal information algorithmically. In brief, 22 participants exercised on nine different resistance exercise machines and performed two times ten repetitions with their respective 60% 1-RM using a user-determined velocity. Smartphones recorded the accelerations exerted on the weight stack. Using these recordings, 99.8% of the repetitions were correctly identified while temporal errors for single repetition time-under-tension (TUT), concentric contraction TUT, eccentric contraction TUT and total TUT were 0.1%, 7.1%, 4.1% and 0.5%, respectively. Although the study design mimicked a real-world scenario by allowing user-determined velocities, slow- or fast-movement repetition velocities with lower or higher intensities were not tested. Therefore, this work aimed to examine the proposed algorithmic behaviour by collecting acceleration data from slow- and fast-movement repetition velocities using low and high intensities reflecting the interindividual variability during resistance exercise. We hypothesized that neither exercise intensity nor movement velocity will disrupt the algorithmic temporal detection.

## Material and methods

### Ethics statement

The study has been approved by the ethics committee of the Swiss Federal Institute of Technology Zurich (ETH Zurich, Zurich, Switzerland) and conducted following the Declaration of Helsinki.

All participants received oral and written information about all procedures of the study and signed a written informed consent.

### Design

This work investigated whether mechano-biological descriptors, *i*.*e*. the number of repetitions, the temporal distribution of contraction modes and total TUT could be extracted from accelerometer derived slow- and fast-movement velocity repetitions of dynamic resistance exercise data performed on a single-joint (leg extension) and a multi-joint (leg press) resistance exercise machine at high and low exercise intensities. Two resistance exercise machines were selected at the Kieser Training AG (Kieser Training AG, Zurich, Switzerland). The selected machines comprised the most often chosen exercises in a lower extremity workout and were as follows: Leg Extension and Leg Press (Kieser Training AG, Zurich, Switzerland). Video recordings, which are considered the gold standard, were made for all exercises.

### Participants

Twenty-seven healthy volunteers between the ages of 22 and 70 years were recruited via academic mailing lists, flyers and word-to-mouth. All participants completed a standard health questionnaire before giving written informed consent to participate in the study. The anthropometrical information of participants is depicted in Table 1.

**Table 1:** Anthropometrical information of participants.

	Young (*n* = 24)	Old (*n* = 3)	Total (*n* = 27)	*p value*
**Age [years]**				< 0.001
Mean (SD)	35.3 (10.8)	65.0 (4.6)	38.6 (13.9)	
Range	22–59	61–70	22–70	
**Sex**				0.681
Male	13 (54.2%)	2 (67.7%)	15 (55.6%)	
Female	11 (45.8%)	1 (33.3%)	12 (44.4%)	
**Weight [kg]**				0.904
Mean (SD)	73.9 (19.3)	75.3 (16.0)	74.1 (18.7)	
Range	49–129	59–91	49–129	
**Height [m]**				0.337
Mean (SD)	1.71 (0.1)	1.73 (0.1)	1.71 (0.1)	
Range	1.56–1.86	1.69–1.82	1.56–1.86	
**Experience**				0.260
no	8 (33.3%)	2 (66.7%)	10 (37.0%)	
yes	16 (66.7%)	1 (33.3%)	17 (63.0%)	
**Experience [mt]**				0.253
Mean (SD)	65 (93.0)	1 (1.7)	57.89 (89.9)	
Range	0–360	0–3	0–360	

Young refers to the population younger than 60 while old includes 60 years and older.

### Equipment

Accelerometer data were collected using two Nexus 6P (Huawei Technologies Co., Ltd., Shenzhen, China) smartphones with a built-in 3-axis accelerometer BMI160 (Robert Bosch GmbH, Stuttgart, Germany).

Two 3D-printed containers served as smartphone holders. The holders were firmly attached to the weight stack using four strong neodymium magnets (Webcraft AG, Uster, Switzerland). During the exercises, the magnet-equipped smartphone holders were attached to the weight stacks of the resistance exercise machines.

### Exercises

Before starting with the measurements, correct settings and range of motion were determined according to participant’s individual anatomy. The participants were familiarized with the motor tasks to be performed on both resistance exercise machines. Next, the participants underwent a ten minute warm-up consisting of climbing stairs.

**Fig 1:**
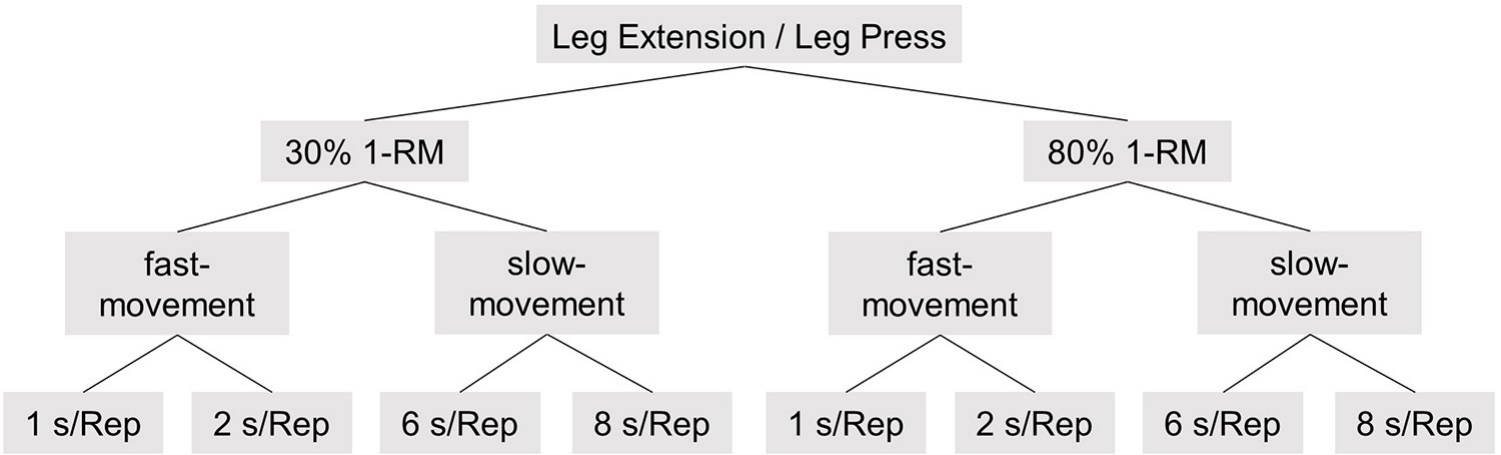
Study design. Two different intensities (30% and 80% one repetition maximum) with fast- (1 s/repetition, 2 s/repetition) and slow-movement repetition (6 s/repetition, 8 s/repetition) velocities on single-joint (leg extension) and multi-joint (leg press) machines were examined.

After the warm-up, the one repetition maximum (1-RM) was determined submaximally. Briefly, participants were asked to choose a resistance level they thought they could lift ten times maximally. Before starting the 1-RM assessment, participants were instructed to lift over the full range of motion. Only repetitions fulfilling this criterion were counted. If the chosen resistance was lifted was more than four but less than ten times, 1-RM was extrapolated, using the formula described in Mayhew *et al*. [18]. If more than ten repetitions were achieved, the exercise was repeated with 20% increase of resistance, following a two-minute recovery break. This was repeated until the number of repetitions was in the defined range. After the 1-RM determination, the volunteers were familiarized with the four velocities using a metronome. Participants then started with the leg press or the leg extension with 30% or 80% of their 1-RM respectively as intensity and exercises were randomized. The protocol consisted of three repetitions for the fast-movement (1 s/Repetition, 2 s/Repetition) and slow-movement (6 s/Repetition, 8 s/Repetition) velocities, for both intensities as depicted in Fig 1. Participants were asked to follow the metronome as closely as possible to ensure repetition duration.

All exercises were recorded with a 62 mm lens Sony HDR-CX900E (Sony, Tokio, Japan) on a tripod using a resolution of 1280 x720 pixels at 25 frames per second. Hence, the sampling frequency between smartphone accelerometer derived measurements and video recordings were different (400 Hz *versus* 25 Hz). However, we do not consider this discrepancy to be a limitation, because method-comparison studies with handheld devices *versus* machines, *e*.*g*. in dynamometry, will never be able to achieve synchronization nor sampling frequency equality [19].

### Rating

#### Video recordings

The free software Kinovea V0.8.27 (www.kinovea.org) was used for reviewing and rating the video recordings. Kinovea is a video player that is widely used for sports analysis. It allows frame-by-frame playback and includes a stopwatch function, which allows for precise annotation of specific time-critical events such as contraction-phases.

**Fig 2:**
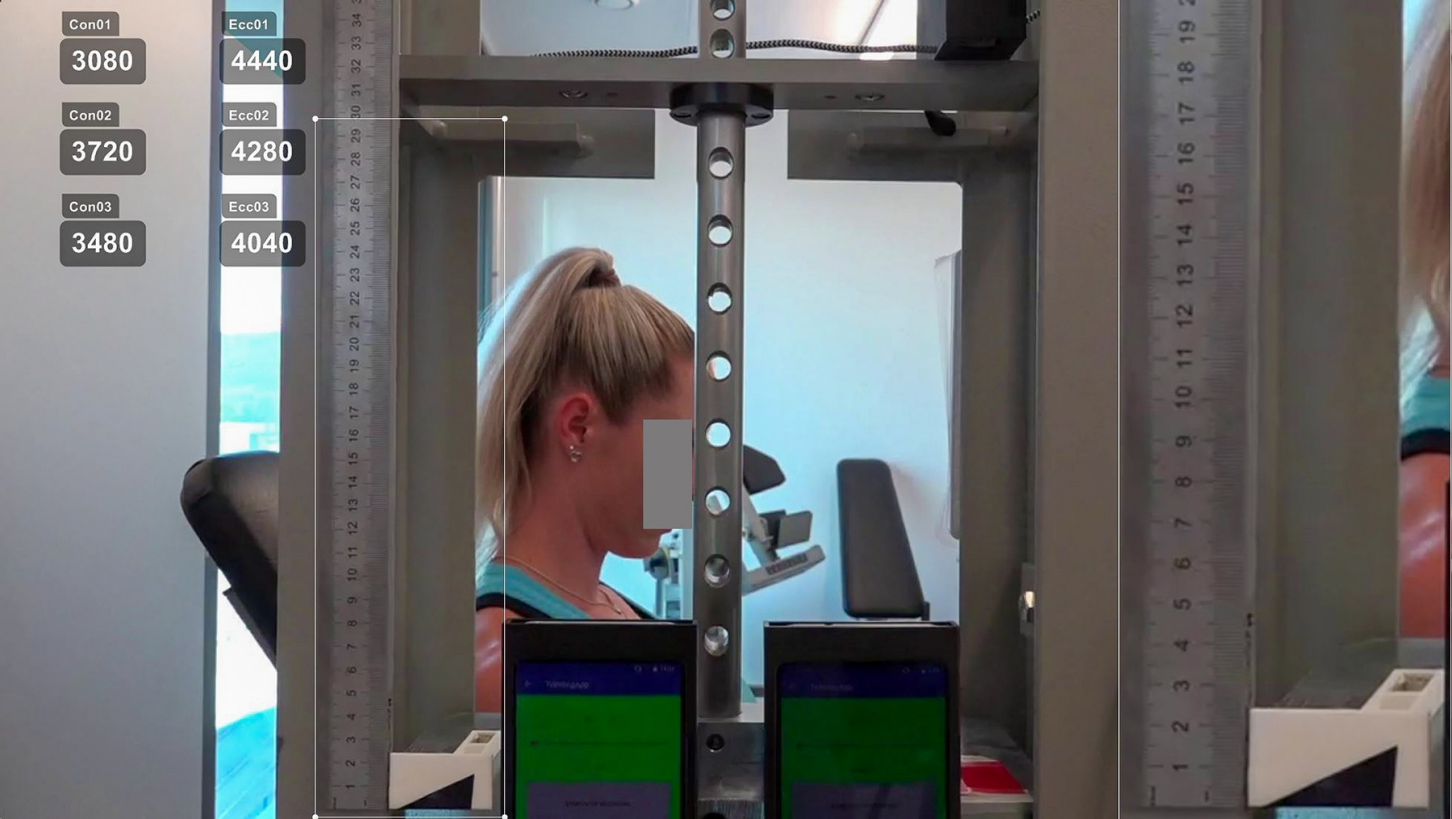
Rating of video recordings. Time per contraction phase was annotated in milliseconds using a 2.5x magnification of the weight stack.

Video recordings were rated by the two study investigators, who screened all recordings independently, frame-by-frame. A 2.5-fold magnification of the weight stack within Kinovea was used to determine contraction phases. The starting point of a concentric contraction was determined as the last frame before the weight stack movement was visually detected. The end of the concentric phase was defined as the first frame, whereby no additional displacement increase of the weight stack could be visually recognized. This frame, due to the dynamic nature of the exercise, was then selected as the starting point of the eccentric phase. The endpoint of the eccentric phase was set to the last frame before the opposite weight stack movement was noticeable. All ten repetitions (20 contraction phases) were annotated in milliseconds in Kinovea as depicted in Fig 2.

#### Smartphone accelerometer derived data

Smartphone accelerometer derived data were analyzed using the algorithm from Viecelli *et al*. [17]. In brief, the vector length was used, and data were pre-processed by applying a Hampel filter to remove outliers [20]. Non-unique timestamps were removed, and the data were subjected to interpolation to achieve an equidistant time series. The gravitational offset was subtracted. Repetition counting was performed by the single integration of the time series. The resulting drift was compensated by a polynomial fit. A moving average filter ensured curve smoothness. Thresholds for minimum inter-repetition distance and prominence were defined for peak detection on the integrated time series. Contraction-specific TUT was determined using the velocity curve zero-crossings.

The following mechano-biological descriptors were extracted from the video recordings and accelerometer data: (I) the number of single repetitions, (II) contraction-specific phases TUT, (III) temporal length of single repetitions as the sum of the concentric and eccentric phase TUT and (IV) the total TUT, which is defined as the sum of all repetitions TUT (3) during a set [16].

### Data and statistical analyses

#### Validity

The analysis aimed to determine whether slow- and/or fast-movement repetition velocities at different intensities would disrupt the algorithmic extraction of mechano-biological resistance exercise descriptors such as the number of single repetitions, contraction-specific phases TUT and the total TUT. Both raters examined video recordings independently and in a randomized order. For the method comparison, the mean of the video recording results and the mean of the algorithmic detection of the two smartphones derived accelerometer data were calculated.

Bland-Altman plots were used to compare the two methods visually. Systematic bias is depicted by the mean difference between the two methods. To examine the linear association between the methods, Pearson correlation coefficients were calculated. Limits of agreement (LoA) were used to determine the level of agreement between methods [21]. The LoA for all contraction phases was calculated as the mean difference between methods, whereby 2.5% or 97.5% denoted the lower and upper limits, respectively [22]. The normalized error was calculated as the division of the contraction-specific mean of the differences between the two methods and the contraction-specific TUT of the algorithmic rating [21].

Methodological outlier removal was performed as described for exploratory studies in [23]. To summarize, the interquartile range (IQR) of the mean difference of the two methods was calculated per contraction-specific phase for both resistance exercise machines. Data values higher than 1.5 or smaller than -1.5 times the IQR were marked and excluded, as suggested by Sachs and Hedderich [23]. Visual assessment of heteroscedasticity was performed without recognizing trends towards heteroscedasticity.

#### Scoring reliability of the two raters

Interrater reliability and agreement were examined between the two raters who rated all 16 sets, consisting of three repetitions each, on two resistance exercises machines of 27 participants. The raters annotated all TUT of all contraction-specific phases. Interrater reliability was calculated using a two-way random-effects model (2.1), single measures, absolute agreement and ICC.

## Results

The detection of single repetitions algorithmically-derived from the two smartphone accelerometers yielded high precision, recall and accuracy. The mean precision was 0.962 ± 0.011 (mean ± SD) for both smartphones. The average accuracy, calculated by the F-Score, for all the exercise machines, was 0.981 ± 0.001 (mean ± SD) equalling an error rate of 1.9%.

**Fig 3:**
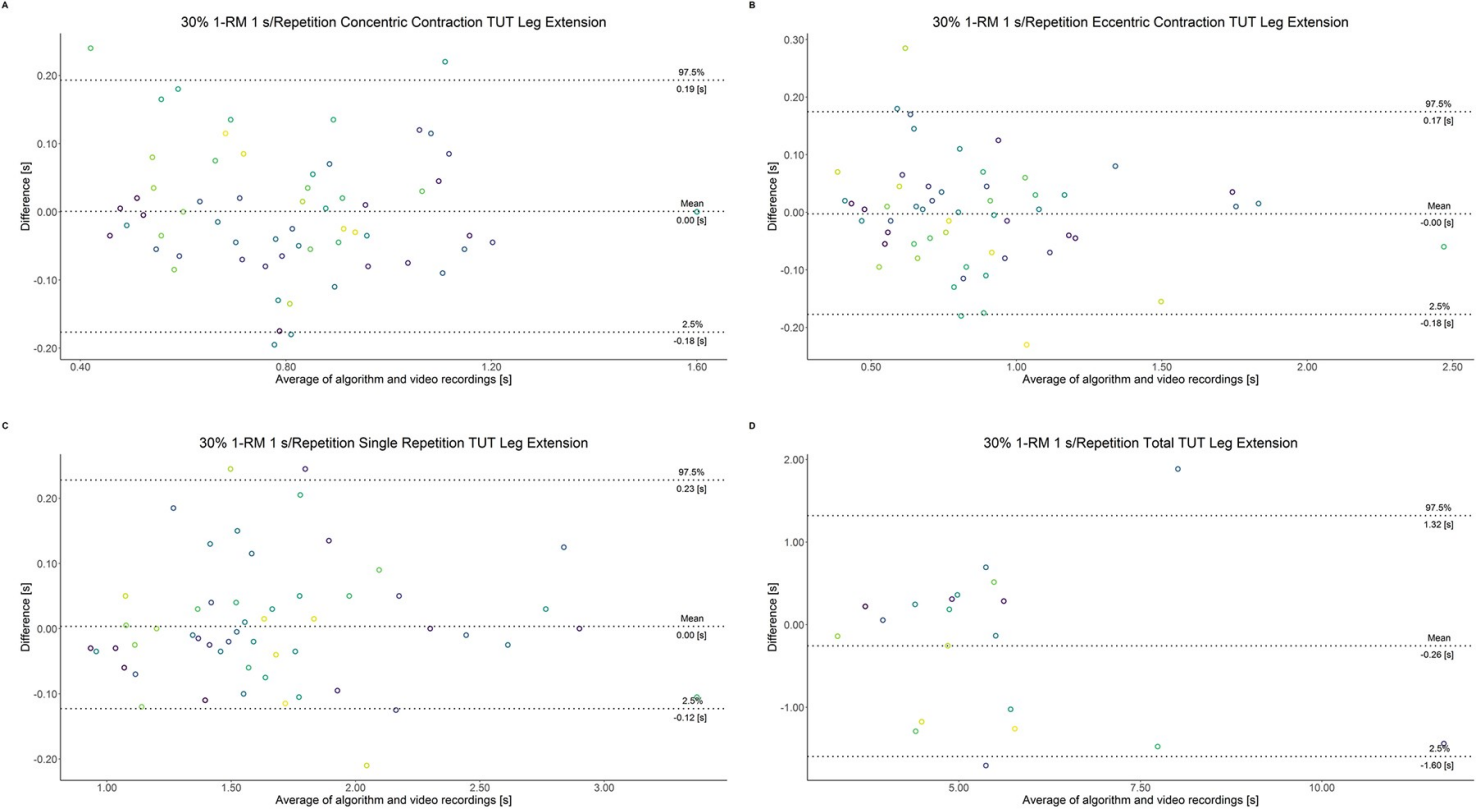
Bland-Altman plots of agreement between algorithmically accelerometer derived and video recordings derived contraction-specific phases of the leg extension machine at 30% 1-RM with a velocity of 1 s per repetition. A: Concentric contraction phase. B: Eccentric contraction phase. C: Single repetition. D: Total time-under-tension.

**Fig 4:**
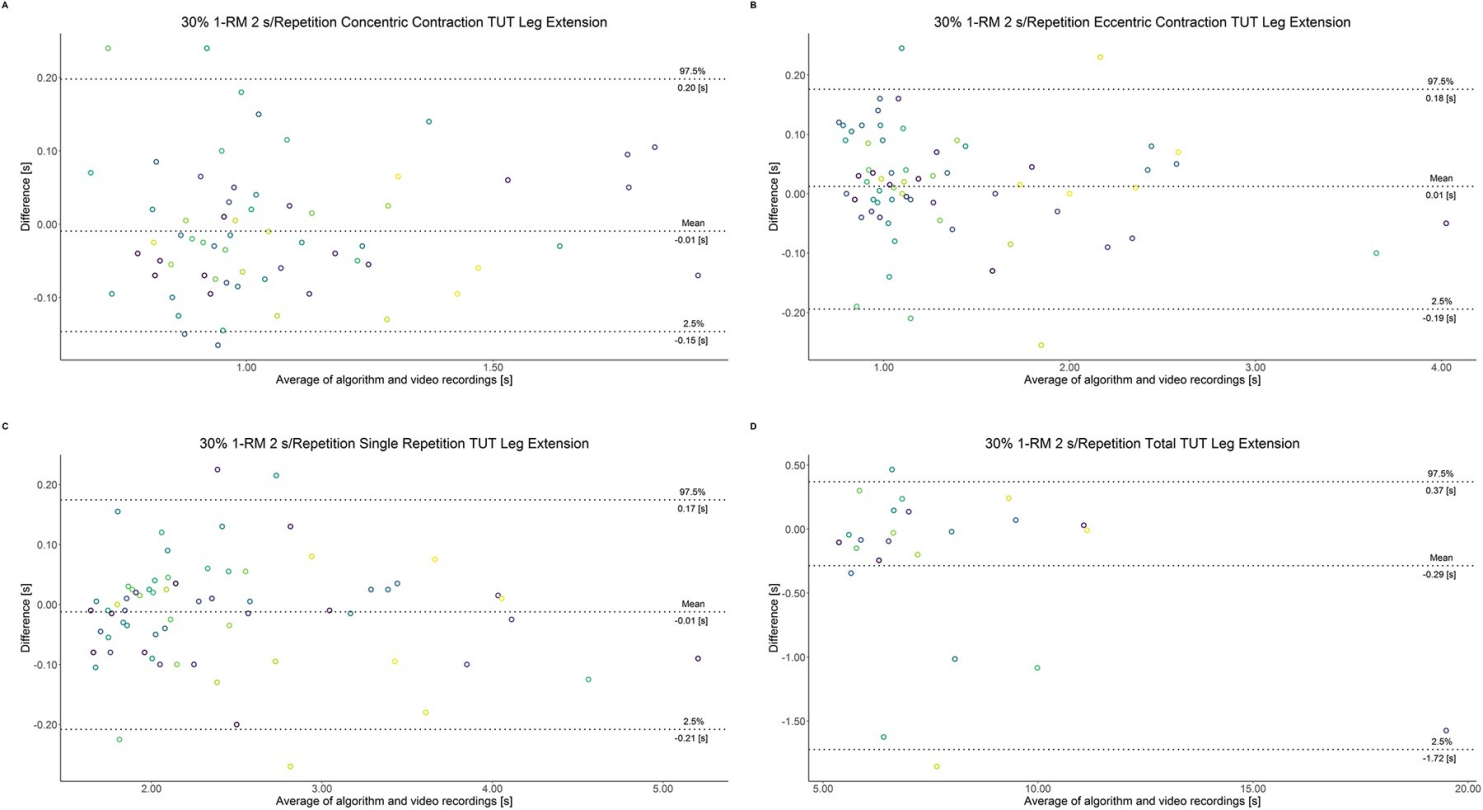
Bland-Altman plots of agreement between algorithmically accelerometer derived and video recordings derived contraction-specific phases of the leg extension machine at 30% 1-RM with a velocity of 2 s per repetition. A: Concentric contraction phase. B: Eccentric contraction phase. C: Single repetition. D: Total time-under-tension.

**Fig 5:**
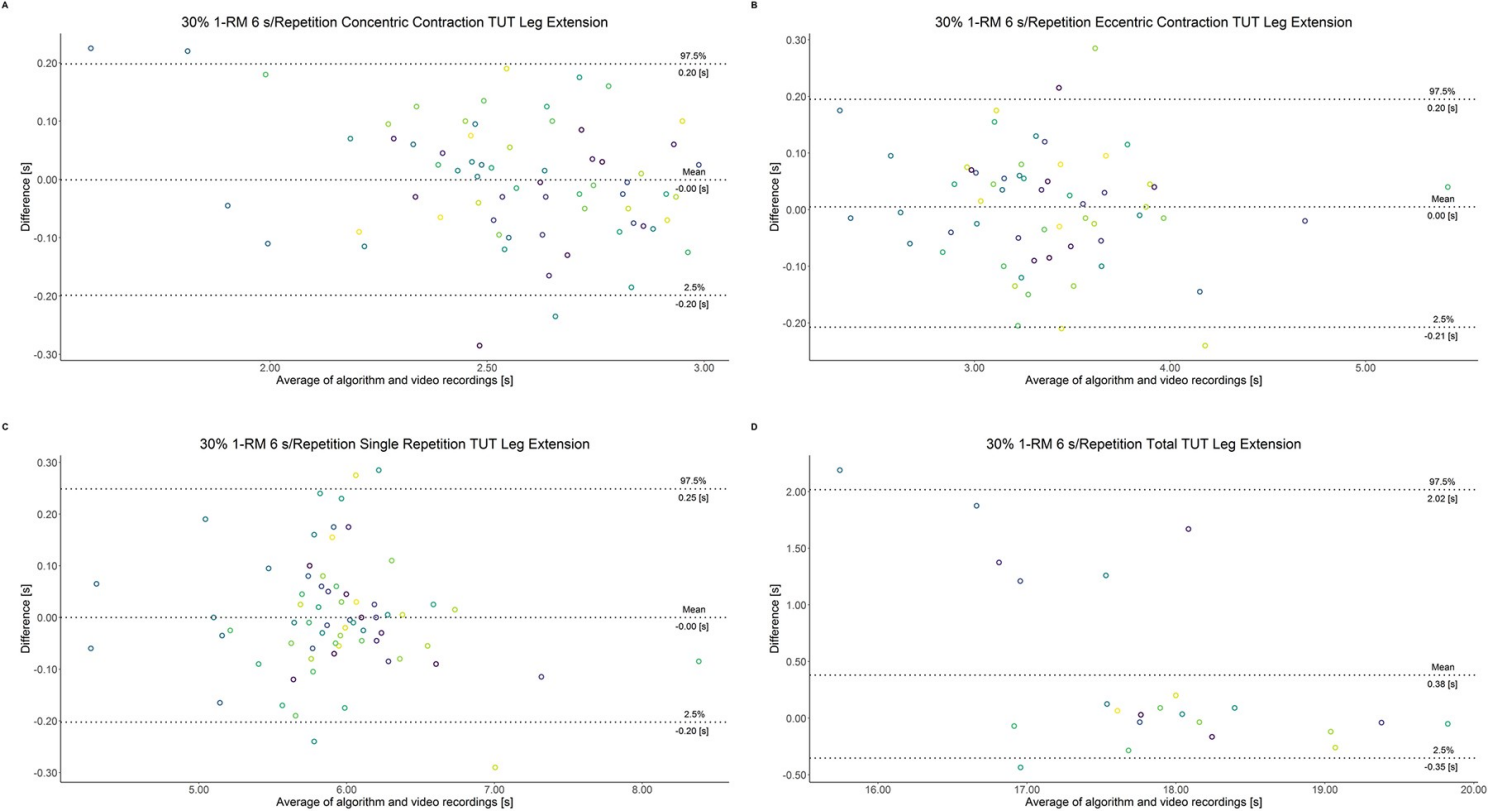
Bland-Altman plots of agreement between algorithmically accelerometer derived and video recordings derived contraction-specific phases of the leg extension machine at 30% 1-RM with a velocity of 6 s per repetition. A: Concentric contraction phase. B: Eccentric contraction phase. C: Single repetition. D: Total time-under-tension.

**Fig 6:**
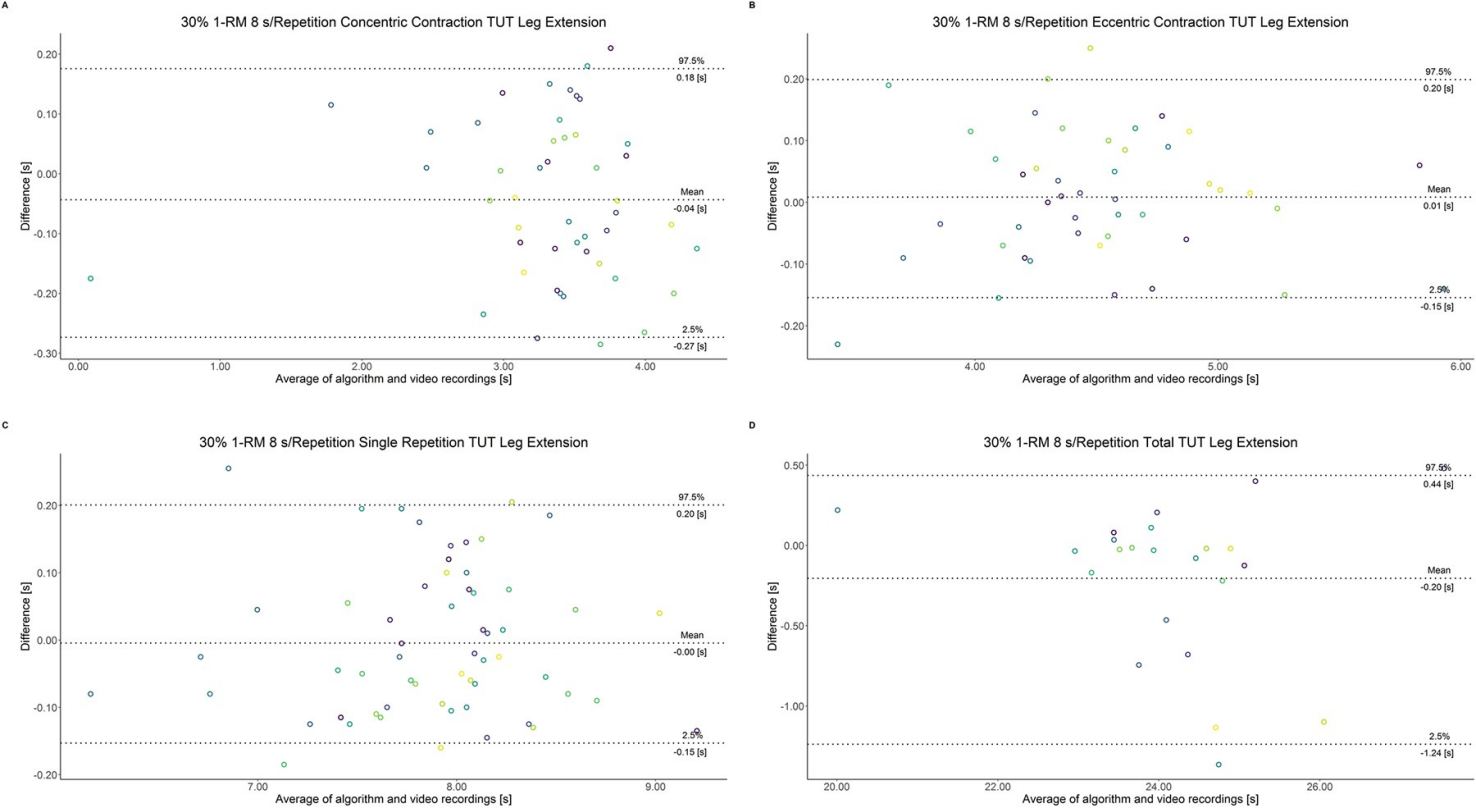
Bland-Altman plots of agreement between algorithmically accelerometer derived and video recordings derived contraction-specific phases of the leg extension machine at 30% 1-RM with a velocity of 8 s per repetition. A: Concentric contraction phase. B: Eccentric contraction phase. C: Single repetition. D: Total time-under-tension.

**Fig 7:**
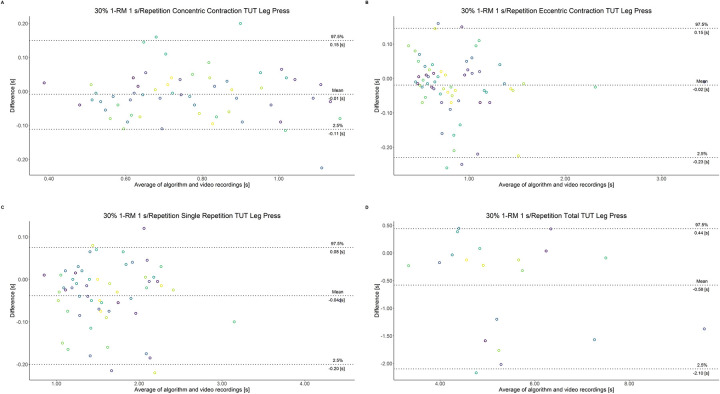
Bland-Altman plots of agreement between algorithmically accelerometer derived and video recordings derived contraction-specific phases of the leg press machine at 30% 1-RM with a velocity of 1 s per repetition. A: Concentric contraction phase. B: Eccentric contraction phase. C: Single repetition. D: Total time-under-tension.

**Fig 8:**
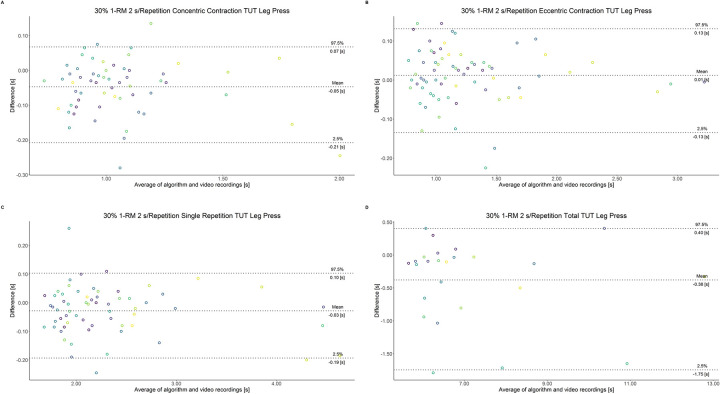
Bland-Altman plots of agreement between algorithmically accelerometer derived and video recordings derived contraction-specific phases of the leg press machine at 30% 1-RM with a velocity of 2 s per repetition. A: Concentric contraction phase. B: Eccentric contraction phase. C: Single repetition. D: Total time-under-tension.

**Fig 9:**
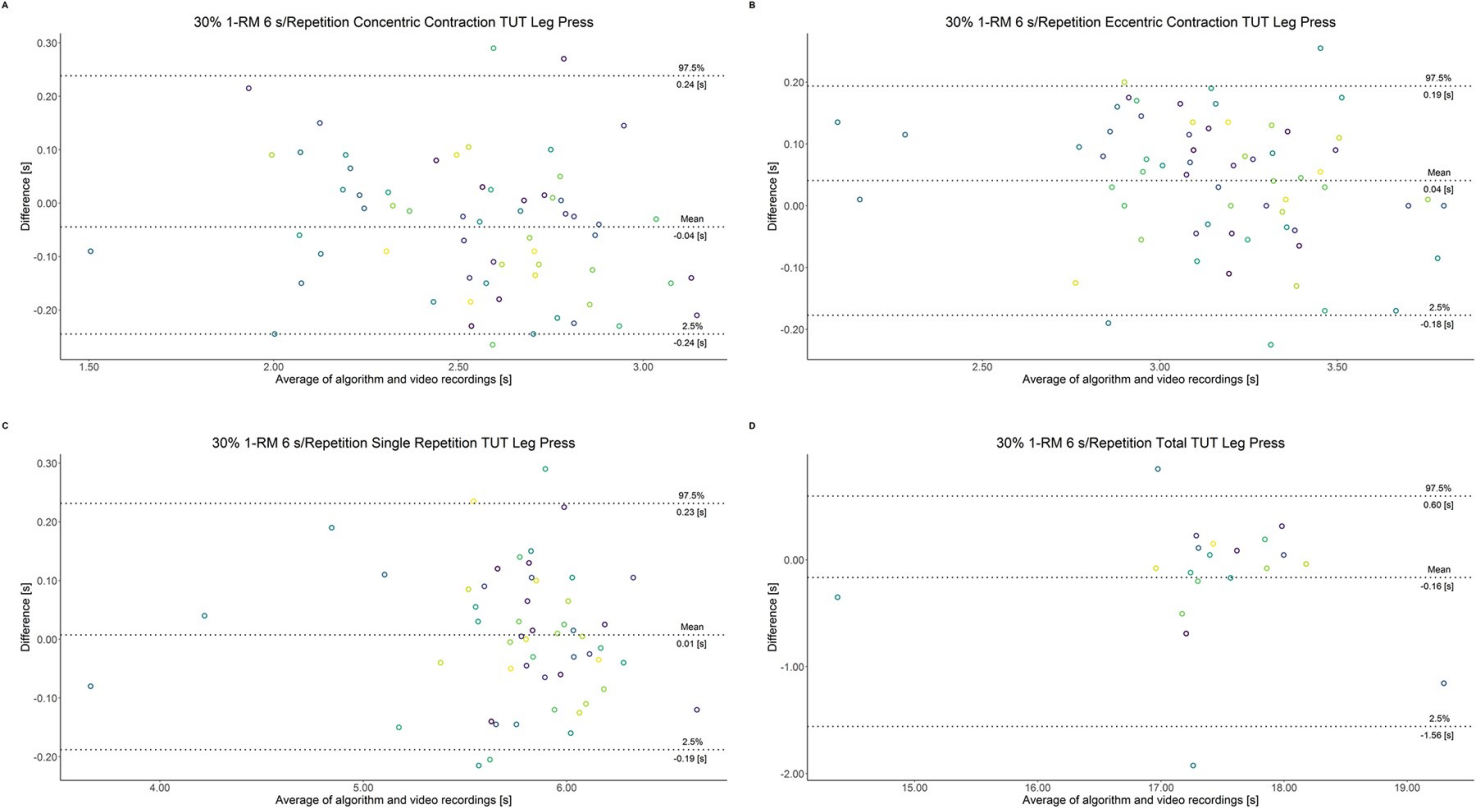
Bland-Altman plots of agreement between algorithmically accelerometer derived and video recordings derived contraction-specific phases of the leg press machine at 30% 1-RM with a velocity of 6 s per repetition. A: Concentric contraction phase. B: Eccentric contraction phase. C: Single repetition. D: Total time-under-tension.

**Fig 10:**
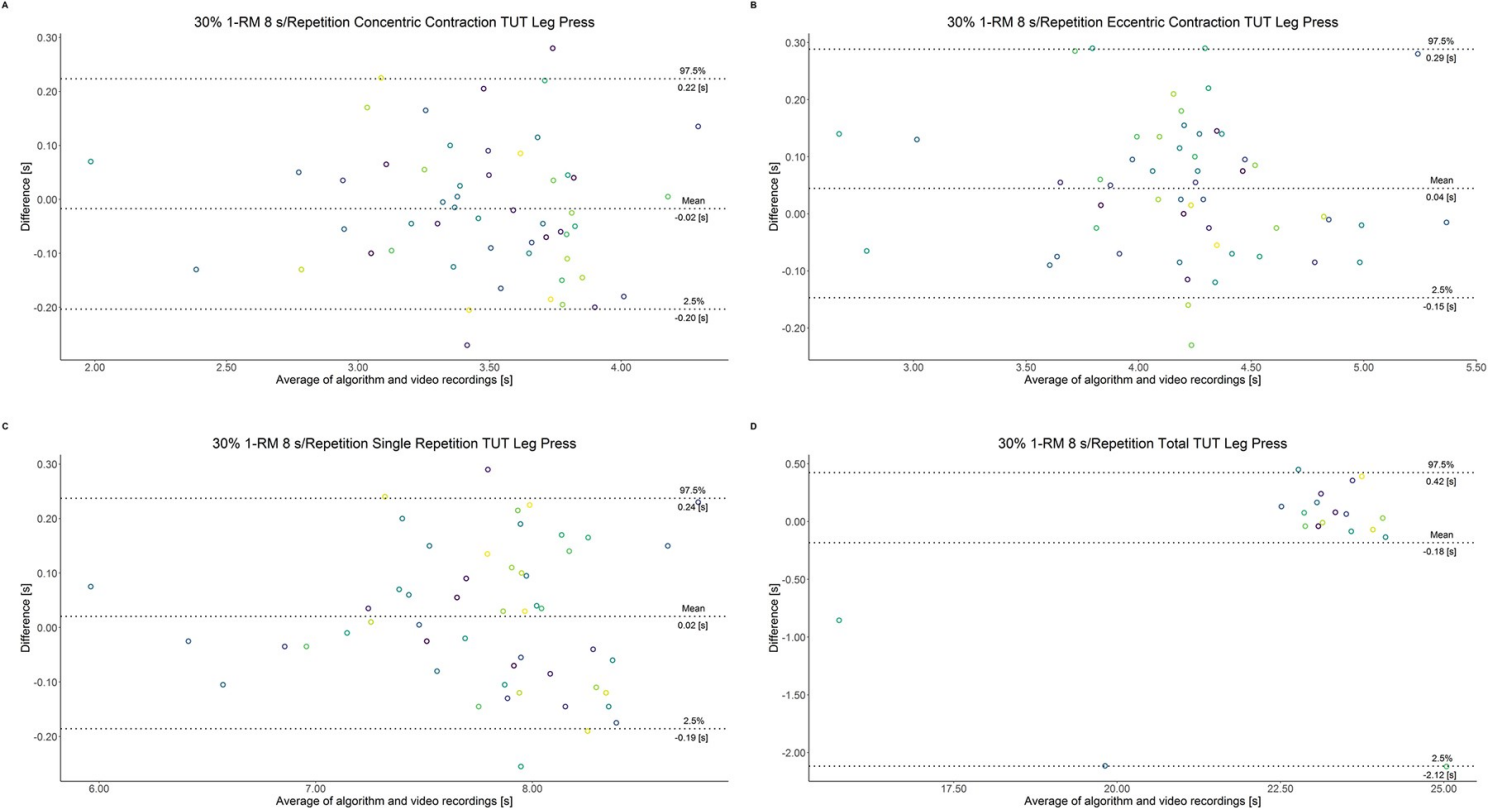
Bland-Altman plots of agreement between algorithmically accelerometer derived and video recordings derived contraction-specific phases of the leg press machine at 30% 1-RM with a velocity of 8 s per repetition. A: Concentric contraction phase. B: Eccentric contraction phase. C: Single repetition. D: Total time-under-tension.

**Fig 11:**
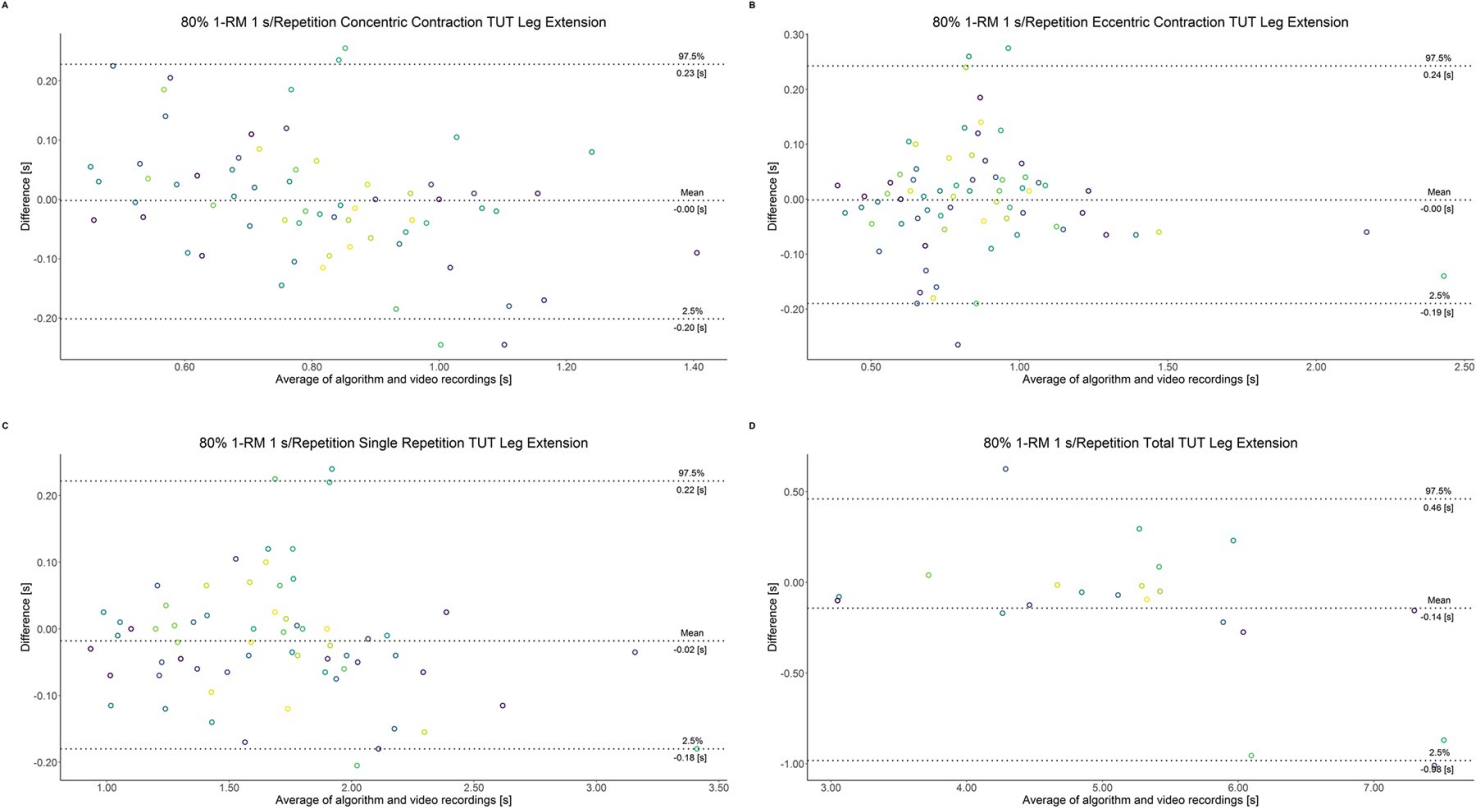
Bland-Altman plots of agreement between algorithmically accelerometer derived and video recordings derived contraction-specific phases of the leg extension machine at 80% 1-RM with a velocity of 1 s per repetition. A: Concentric contraction phase. B: Eccentric contraction phase. C: Single repetition. D: Total time-under-tension.

**Fig 12:**
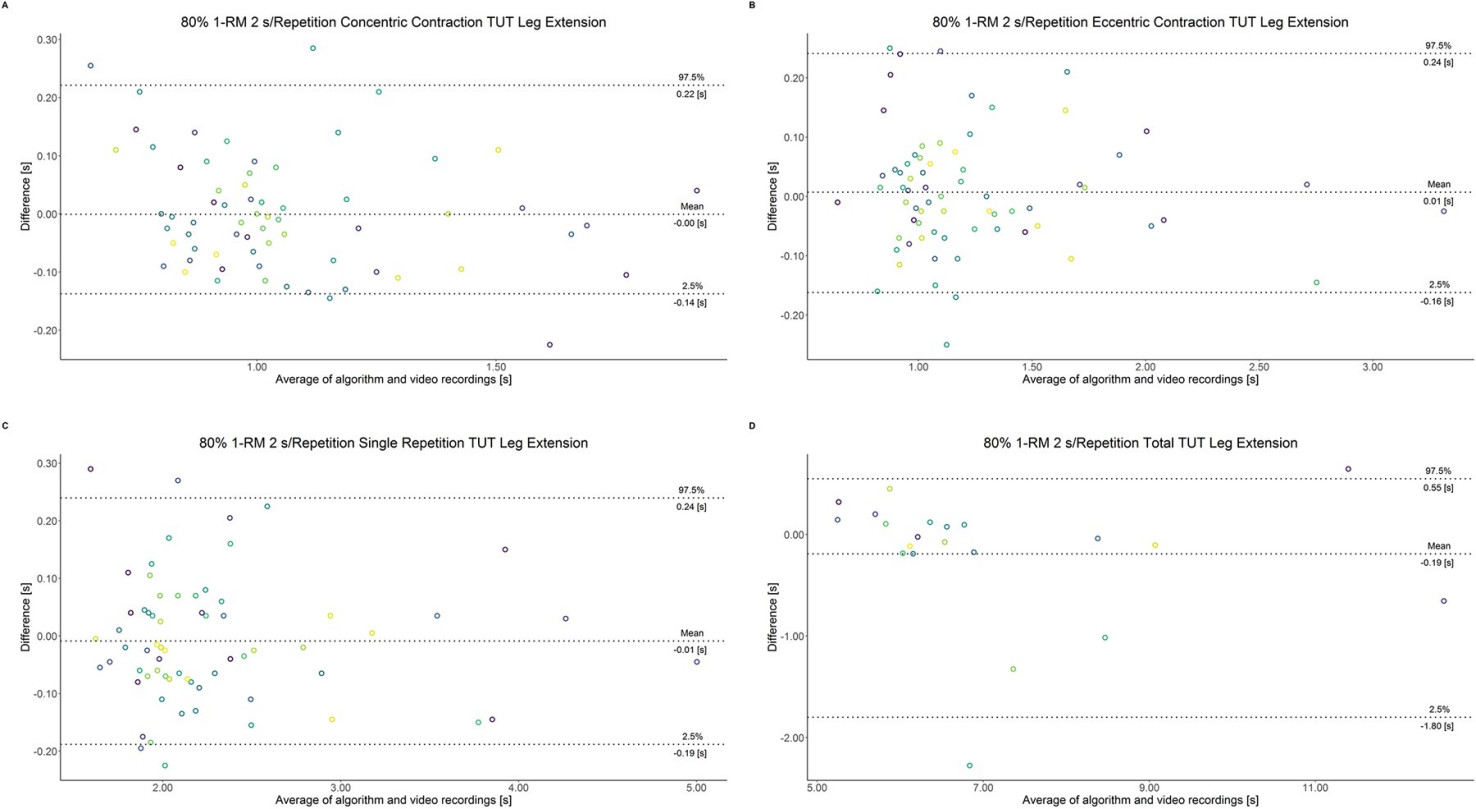
Bland-Altman plots of agreement between algorithmically accelerometer derived and video recordings derived contraction-specific phases of the leg extension machine at 80% 1-RM with a velocity of 2 s per repetition. A: Concentric contraction phase. B: Eccentric contraction phase. C: Single repetition. D: Total time-under-tension.

**Fig 13:**
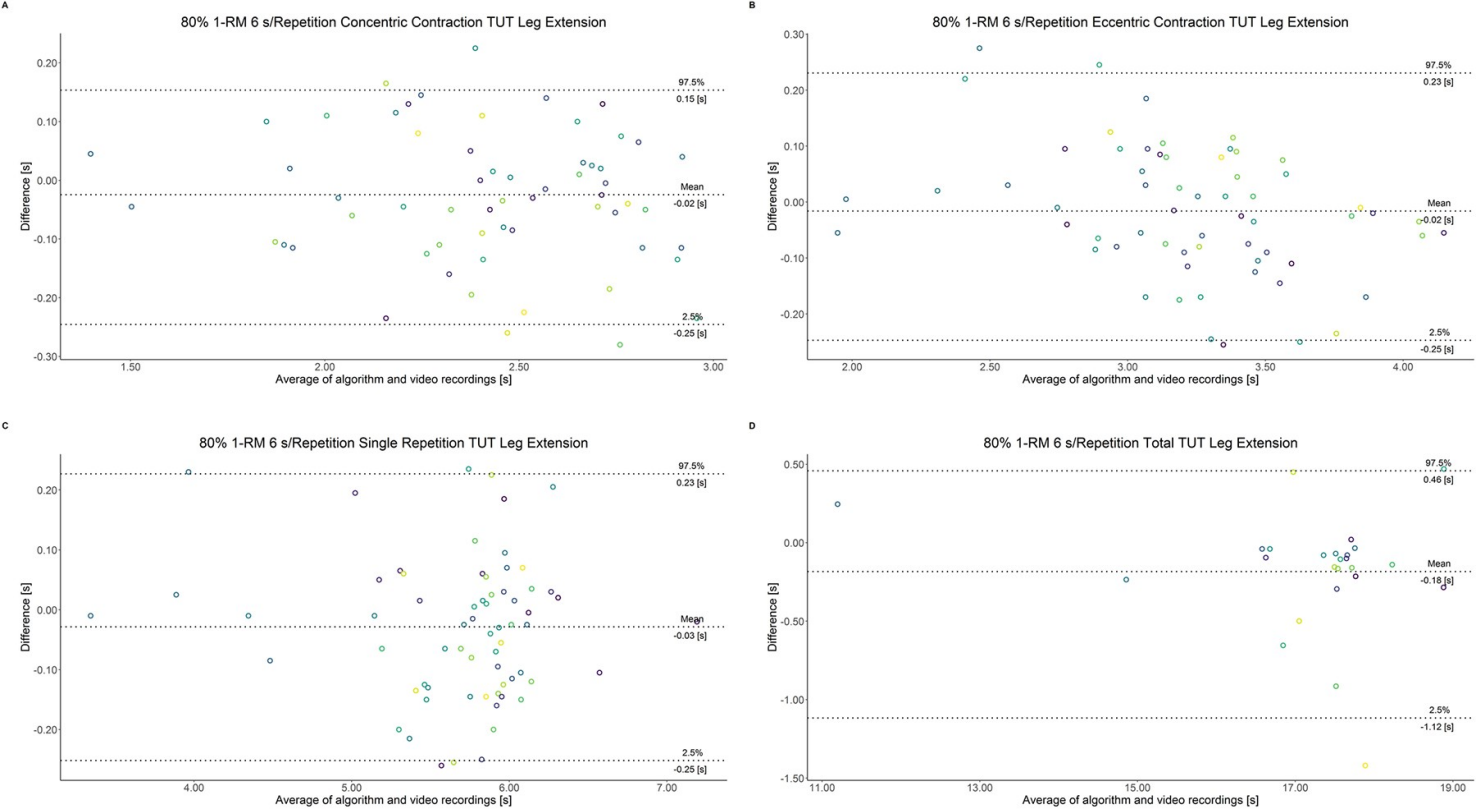
Bland-Altman plots of agreement between algorithmically accelerometer derived and video recordings derived contraction-specific phases of the leg extension machine at 80% 1-RM with a velocity of 6 s per repetition. A: Concentric contraction phase. B: Eccentric contraction phase. C: Single repetition. D: Total time-under-tension.

**Fig 14:**
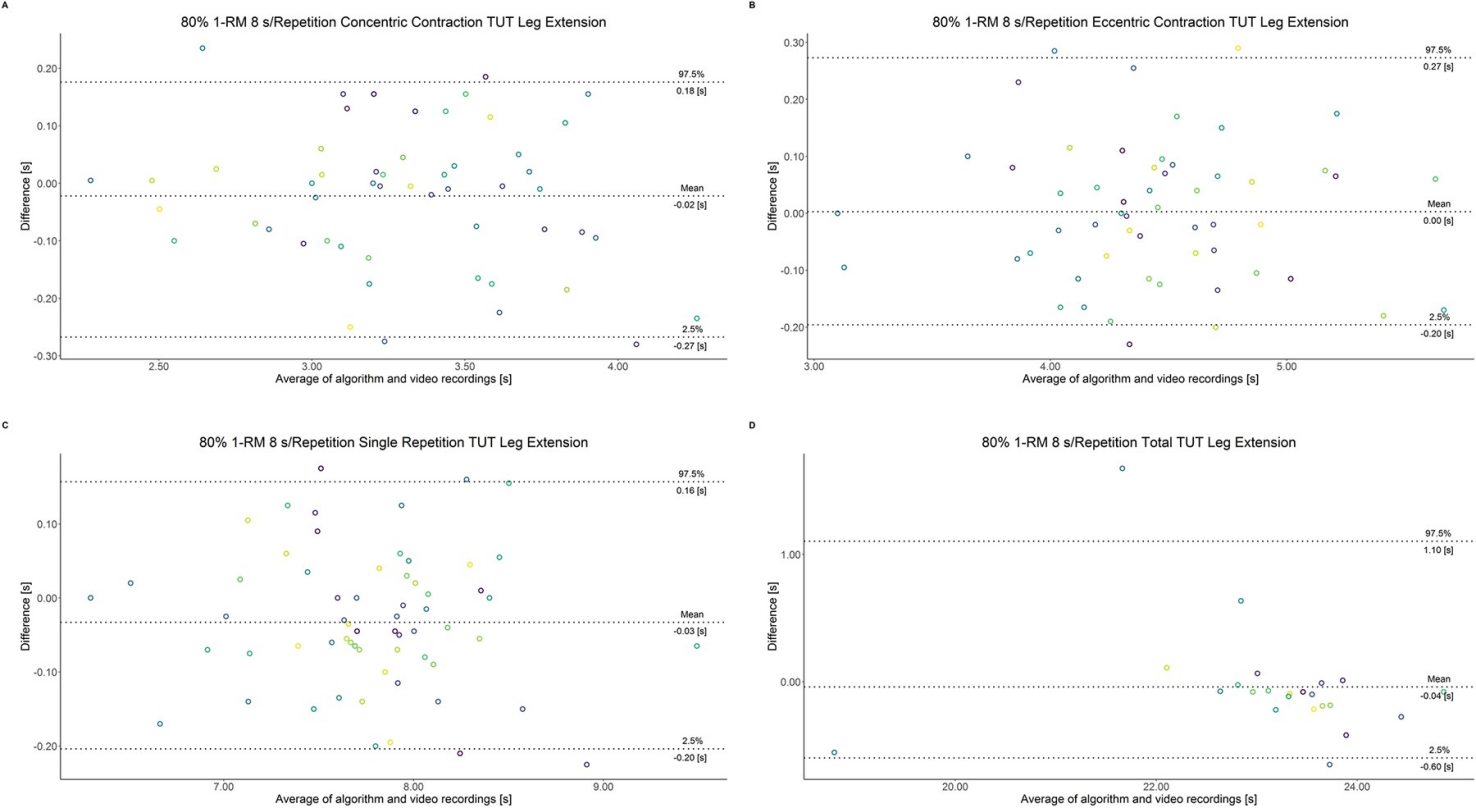
Bland-Altman plots of agreement between algorithmically accelerometer derived and video recordings derived contraction-specific phases of the leg extension machine at 80% 1-RM with a velocity of 8 s per repetition. A: Concentric contraction phase. B: Eccentric contraction phase. C: Single repetition. D: Total time-under-tension.

**Fig 15:**
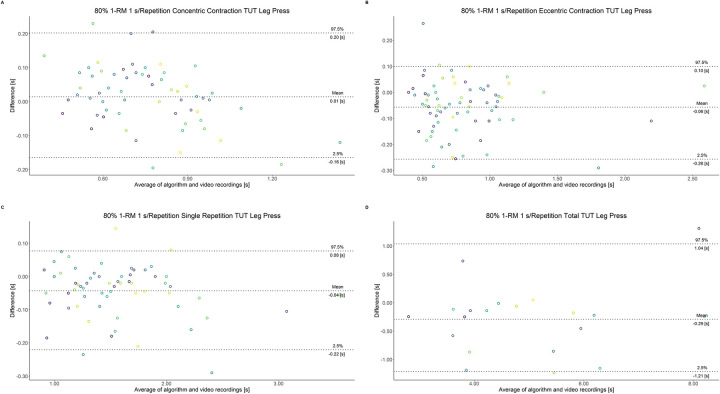
Bland-Altman plots of agreement between algorithmically accelerometer derived and video recordings derived contraction-specific phases of the leg press machine at 80% 1-RM with a velocity of 1 s per repetition. A: Concentric contraction phase. B: Eccentric contraction phase. C: Single repetition. D: Total time-under-tension.

**Fig 16:**
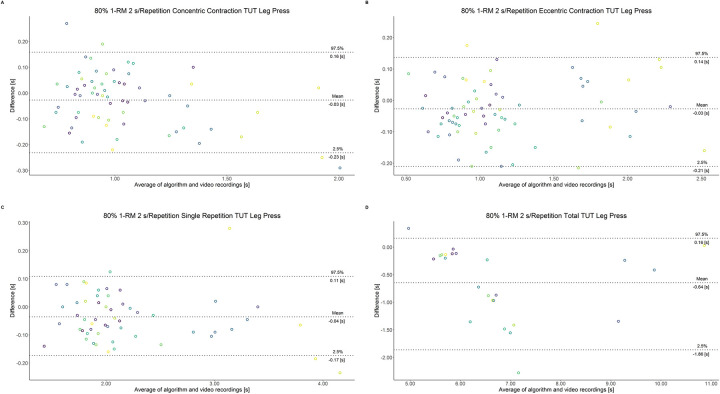
Bland-Altman plots of agreement between algorithmically accelerometer derived and video recordings derived contraction-specific phases of the leg press machine at 80% 1-RM with a velocity of 2 s per repetition. A: Concentric contraction phase. B: Eccentric contraction phase. C: Single repetition. D: Total time-under-tension.

**Fig 17:**
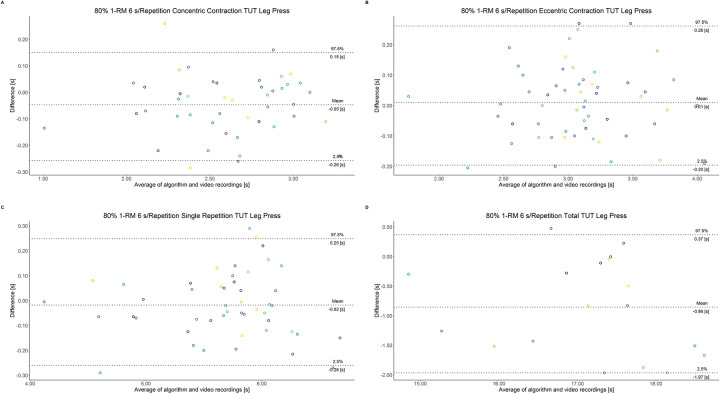
Bland-Altman plots of agreement between algorithmically accelerometer derived and video recordings derived contraction-specific phases of the leg press machine at 80% 1-RM with a velocity of 6 s per repetition. A: Concentric contraction phase. B: Eccentric contraction phase. C: Single repetition. D: Total time-under-tension.

**Fig 18:**
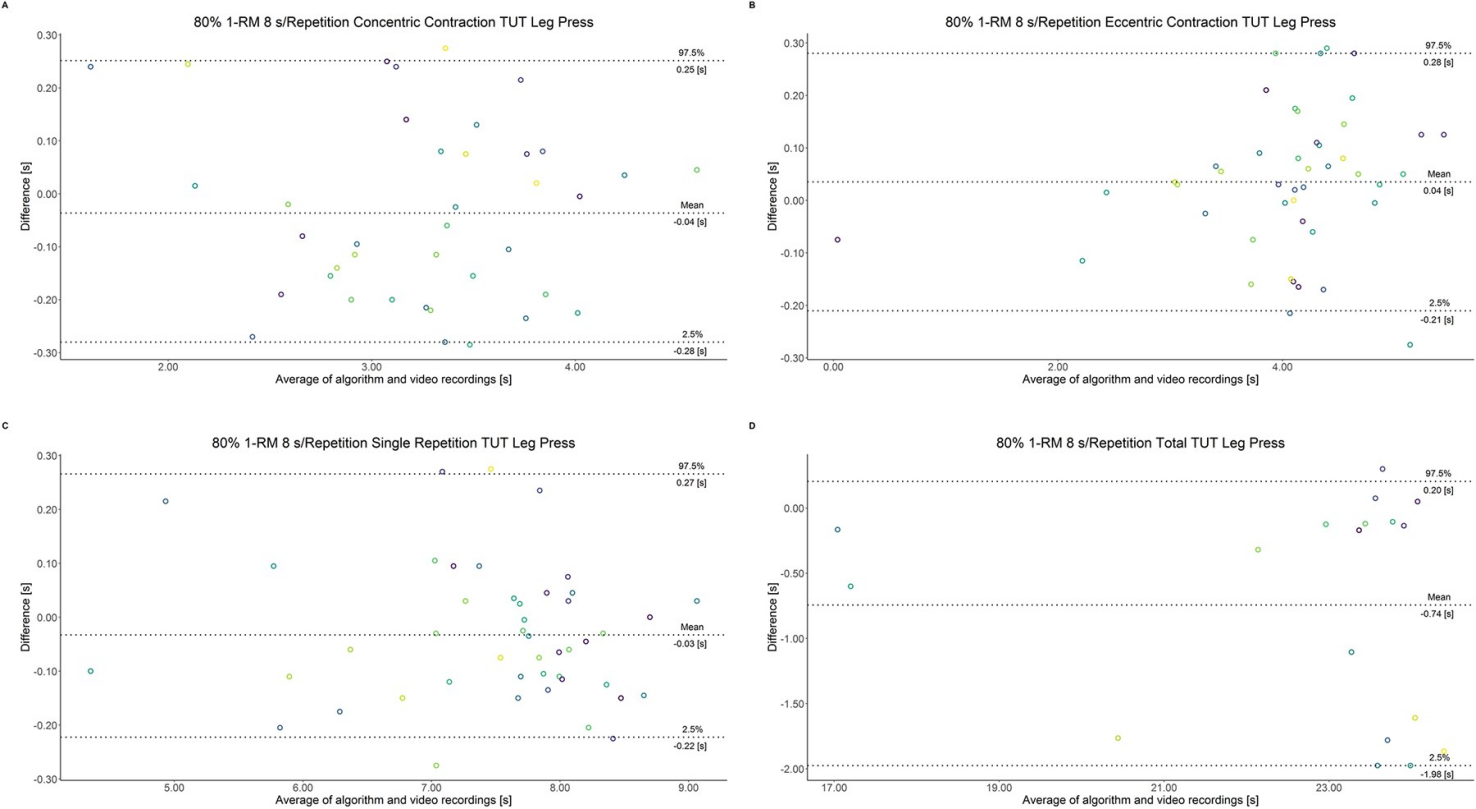
Bland-Altman plots of agreement between algorithmically accelerometer derived and video recordings derived contraction-specific phases of the leg press machine at 80% 1-RM with a velocity of 8 s per repetition. A: Concentric contraction phase. B: Eccentric contraction phase. C: Single repetition. D: Total time-under-tension.

**Fig 19:**
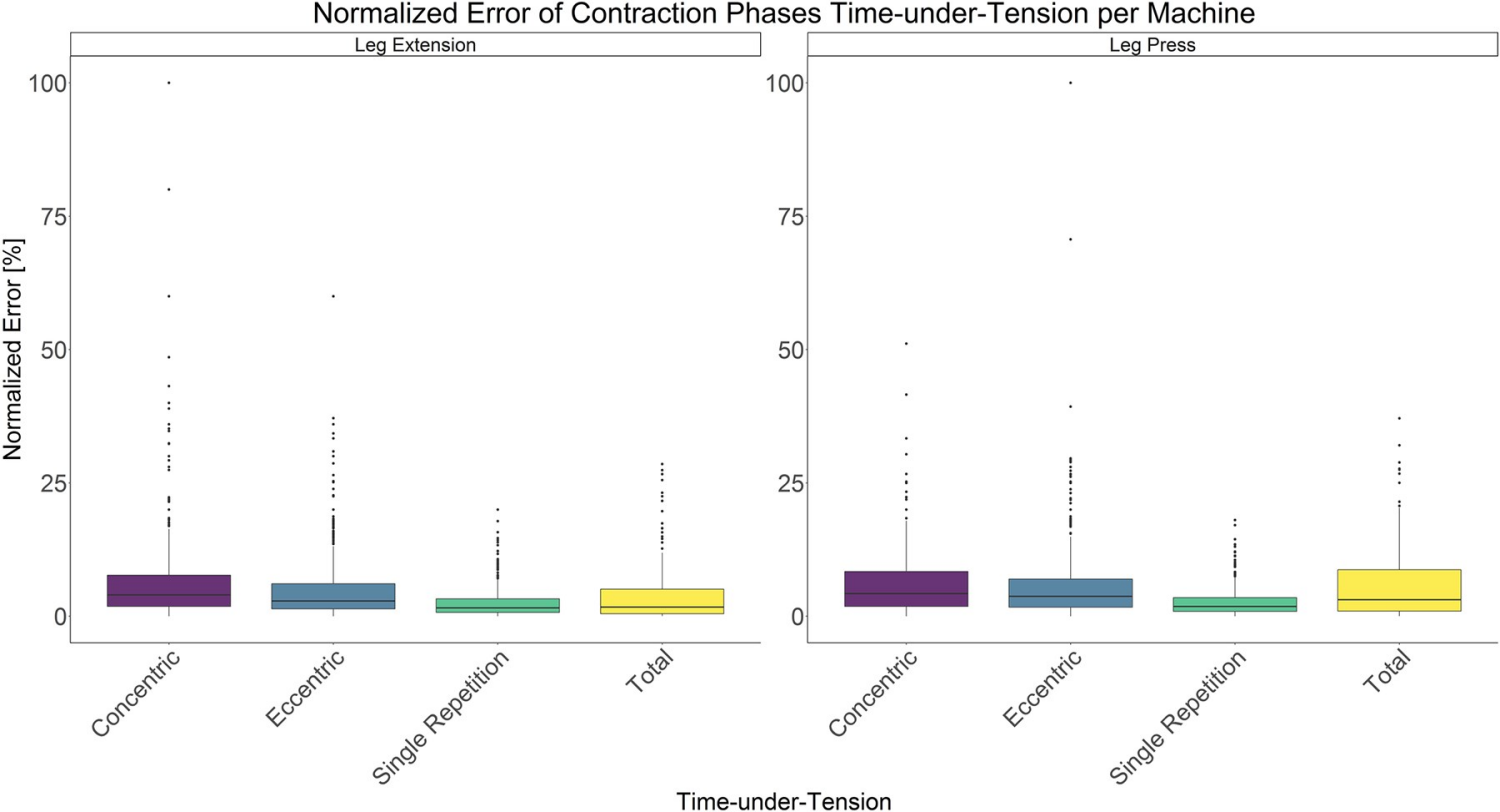
Boxplot of normalized errors of contraction-specific phases for both resistance exercise machines.

The comparison between video recordings and the algorithmically-derived, contraction-phase specific TUT, showed a high degree of correlation (r > 0.94) for both exercise machines (Table 2). Besides, the ICC for the interrater reliability was above 0.99 with 95% CI [0.99, 1.00] for all contraction-phase specific TUT. Table 2 depicts the agreement between concentric, eccentric, single repetition and total TUT derived from algorithmic accelerometer data and video recordings. In Figs 3–18 Bland-Altman plots show the systematic bias as the mean difference between the methods whereas Fig 19 visualizes the normalized errors of contraction-specific phases for both resistance exercise machines.

**Table 2:** Agreement between concentric, eccentric, single repetition and total time-under-tension, derived from algorithmic accelerometer data and video recordings.

Intensity [%-1RM]	Machine	Repetition Velocity [s]	Contraction Mode Level	Mean time based on accelerometer data [s] (±SD)	Mean time based on video recordings [s] (±SD)	Mean difference [s] (±SD)	Correlation	95% Limits of Agreement [s]	Agreement (% of mean TUT from algorithm)
30	Leg Extension	1	Concentric TUT (*n =* 68)	0.79 (0.23)	0.8 (0.23)	0 (0.09)	0.93***	-0.18–0.19	9.6
30	Leg Extension	1	Eccentric TUT (*n =* 63)	0.86 (0.38)	0.86 (0.37)	0 (0.09)	0.97***	-0.18–0.17	8.6
30	Leg Extension	1	Single Repetition TUT (*n =* 58)	1.68 (0.51)	1.68 (0.51)	0 (0.09)	0.98***	-0.12–0.23	4.2
30	Leg Extension	1	Total TUT (*n =* 20)	5.64 (1.99)	5.38 (1.82)	0.26 (0.93)	0.88***	-1.6–1.32	11.98
30	Leg Extension	2	Concentric TUT (*n =* 69)	1.07 (0.27)	1.06 (0.28)	0.01 (0.09)	0.95***	-0.15–0.2	7.05
30	Leg Extension	2	Eccentric TUT (*n =* 72)	1.34 (0.65)	1.36 (0.64)	0.01 (0.09)	0.99***	-0.19–0.18	5.67
30	Leg Extension	2	Single Repetition TUT (*n =* 68)	2.46 (0.79)	2.45 (0.78)	0.01 (0.09)	0.99***	-0.21–0.17	2.78
30	Leg Extension	2	Total TUT (*n =* 24)	8 (3.14)	7.71 (2.89)	0.29 (0.64)	0.98***	-1.72–0.37	4.97
30	Leg Extension	6	Concentric TUT (*n =* 70)	2.56 (0.3)	2.56 (0.27)	0 (0.1)	0.94***	-0.2–0.2	3.32
30	Leg Extension	6	Eccentric TUT (*n =* 61)	3.37 (0.51)	3.37 (0.5)	0 (0.1)	0.98***	-0.21–0.2	2.43
30	Leg Extension	6	Single Repetition TUT (*n =* 71)	5.93 (0.57)	5.93 (0.56)	0 (0.11)	0.98***	-0.2–0.25	1.41
30	Leg Extension	6	Total TUT (*n =* 23)	17.64 (1.21)	18.02 (0.78)	0.38 (0.77)	0.78***	-0.35–2.02	3.12
30	Leg Extension	8	Concentric TUT (*n =* 47)	3.36 (0.68)	3.31 (0.67)	0.04 (0.13)	0.98***	-0.27–0.18	5.46
30	Leg Extension	8	Eccentric TUT (*n =* 45)	4.51 (0.5)	4.51 (0.5)	0.01 (0.11)	0.98***	-0.15–0.2	1.92
30	Leg Extension	8	Single Repetition TUT (*n =* 59)	7.88 (0.54)	7.88 (0.54)	0 (0.11)	0.98***	-0.15–0.2	1.17
30	Leg Extension	8	Total TUT (*n =* 23)	24.29 (1.41)	24.08 (1.34)	0.2 (0.49)	0.94***	-1.24–0.44	1.35
30	Leg Press	1	Concentric TUT (*n =* 68)	0.76 (0.19)	0.75 (0.19)	0.01 (0.07)	0.93***	-0.11–0.15	6.87
30	Leg Press	1	Eccentric TUT (*n =* 73)	0.86 (0.46)	0.84 (0.45)	0.02 (0.08)	0.98***	-0.23–0.15	7.77
30	Leg Press	1	Single Repetition TUT (*n =* 61)	1.66 (0.58)	1.62 (0.57)	0.04 (0.07)	0.99***	-0.2–0.08	3.76
30	Leg Press	1	Total TUT (*n =* 20)	5.71 (1.59)	5.13 (1.39)	0.58 (0.87)	0.84***	-2.1–0.44	11.81
30	Leg Press	2	Concentric TUT (*n =* 71)	1.06 (0.24)	1.01 (0.23)	0.05 (0.07)	0.95***	-0.21–0.07	5.9
30	Leg Press	2	Eccentric TUT (*n =* 77)	1.24 (0.48)	1.25 (0.48)	0.01 (0.07)	0.99***	-0.13–0.13	4.59
30	Leg Press	2	Single Repetition TUT (*n =* 68)	2.32 (0.68)	2.29 (0.67)	0.03 (0.08)	0.99***	-0.19–0.1	2.87
30	Leg Press	2	Total TUT (*n =* 25)	7.35 (1.86)	6.97 (1.77)	0.38 (0.62)	0.94***	-1.75–0.4	6.2
30	Leg Press	6	Concentric TUT (*n =* 64)	2.57 (0.34)	2.53 (0.32)	0.04 (0.13)	0.93***	-0.24–0.24	4.18
30	Leg Press	6	Eccentric TUT (*n =* 66)	3.14 (0.35)	3.18 (0.32)	0.04 (0.1)	0.95***	-0.18–0.19	2.99
30	Leg Press	6	Single Repetition TUT (*n =* 56)	5.76 (0.47)	5.77 (0.47)	0.01 (0.11)	0.97***	-0.19–0.23	1.58
30	Leg Press	6	Total TUT (*n =* 20)	17.5 (0.96)	17.33 (0.92)	0.16 (0.58)	0.81***	-1.56–0.6	2.07
30	Leg Press	8	Concentric TUT (*n =* 54)	3.47 (0.43)	3.46 (0.42)	0.02 (0.12)	0.96***	-0.2–0.22	2.94
30	Leg Press	8	Eccentric TUT (*n =* 54)	4.17 (0.51)	4.21 (0.5)	0.04 (0.12)	0.97***	-0.15–0.29	2.49
30	Leg Press	8	Single Repetition TUT (*n =* 52)	7.76 (0.55)	7.78 (0.55)	0.02 (0.13)	0.97***	-0.19–0.24	1.38
30	Leg Press	8	Total TUT (*n =* 19)	22.93 (1.91)	22.75 (2.14)	0.18 (0.73)	0.94***	-2.12–0.42	1.77
80	Leg Extension	1	Concentric TUT (*n =* 70)	0.81 (0.23)	0.81 (0.19)	0 (0.1)	0.89***	-0.2–0.23	10.17
80	Leg Extension	1	Eccentric TUT (*n =* 76)	0.87 (0.34)	0.87 (0.33)	0 (0.1)	0.96***	-0.19–0.24	8.51
80	Leg Extension	1	Single Repetition TUT (*n =* 66)	1.71 (0.48)	1.69 (0.47)	0.02 (0.09)	0.98***	-0.18–0.22	4
80	Leg Extension	1	Total TUT (*n =* 21)	5.33 (1.37)	5.19 (1.16)	0.14 (0.39)	0.97***	-0.98–0.46	4.51
80	Leg Extension	2	Concentric TUT (*n =* 71)	1.06 (0.27)	1.06 (0.25)	0 (0.1)	0.93***	-0.14–0.22	7.7
80	Leg Extension	2	Eccentric TUT (*n =* 73)	1.23 (0.48)	1.24 (0.47)	0.01 (0.1)	0.98***	-0.16–0.24	6.86
80	Leg Extension	2	Single Repetition TUT (*n =* 68)	2.3 (0.66)	2.29 (0.65)	0.01 (0.11)	0.99***	-0.19–0.24	3.9
80	Leg Extension	2	Total TUT (*n =* 21)	7.22 (1.98)	7.03 (1.89)	0.19 (0.65)	0.94***	-1.8–0.55	5.13
80	Leg Extension	6	Concentric TUT (*n =* 64)	2.44 (0.35)	2.42 (0.33)	0.02 (0.11)	0.95***	-0.25–0.15	3.75
80	Leg Extension	6	Eccentric TUT (*n =* 64)	3.23 (0.47)	3.22 (0.43)	0.02 (0.11)	0.97***	-0.25–0.23	2.88
80	Leg Extension	6	Single Repetition TUT (*n =* 67)	5.7 (0.61)	5.67 (0.6)	0.03 (0.12)	0.98***	-0.25–0.23	1.7
80	Leg Extension	6	Total TUT (*n =* 25)	17.27 (1.52)	17.09 (1.45)	0.18 (0.39)	0.97***	-1.12–0.46	1.6
80	Leg Extension	8	Concentric TUT (*n =* 53)	3.32 (0.44)	3.3 (0.42)	0.02 (0.12)	0.96***	-0.27–0.18	2.83
80	Leg Extension	8	Eccentric TUT (*n =* 57)	4.44 (0.5)	4.44 (0.5)	0 (0.12)	0.97***	-0.2–0.27	2.2
80	Leg Extension	8	Single Repetition TUT (*n =* 66)	7.8 (0.54)	7.77 (0.53)	0.03 (0.09)	0.98***	-0.2–0.16	1.01
80	Leg Extension	8	Total TUT (*n =* 23)	23.16 (1.23)	23.12 (1.15)	0.04 (0.45)	0.93***	-0.6–1.1	1.16
80	Leg Press	1	Concentric TUT (*n =* 64)	0.74 (0.21)	0.76 (0.18)	0.01 (0.09)	0.91***	-0.16–0.2	10.35
80	Leg Press	1	Eccentric TUT (*n =* 79)	0.82 (0.36)	0.77 (0.36)	0.06 (0.1)	0.96***	-0.26–0.1	11.32
80	Leg Press	1	Single Repetition TUT (*n =* 64)	1.58 (0.5)	1.54 (0.49)	0.04 (0.08)	0.99***	-0.22–0.08	4.04
80	Leg Press	1	Total TUT (*n =* 20)	5.11 (1.44)	4.82 (1.56)	0.29 (0.61)	0.92***	-1.21–1.04	9.81
80	Leg Press	2	Concentric TUT (*n =* 66)	1.06 (0.31)	1.03 (0.27)	0.03 (0.11)	0.94***	-0.23–0.16	8.32
80	Leg Press	2	Eccentric TUT (*n =* 75)	1.17 (0.45)	1.15 (0.46)	0.03 (0.09)	0.98***	-0.21–0.14	7.19
80	Leg Press	2	Single Repetition TUT (*n =* 59)	2.25 (0.65)	2.21 (0.63)	0.04 (0.09)	0.99***	-0.17–0.11	3.41
80	Leg Press	2	Total TUT (*n =* 24)	7.14 (1.59)	6.5 (1.48)	0.64 (0.66)	0.91***	-1.86–0.16	9.09
80	Leg Press	6	Concentric TUT (*n =* 47)	2.64 (0.36)	2.59 (0.36)	0.05 (0.11)	0.95***	-0.26–0.15	3.55
80	Leg Press	6	Eccentric TUT (*n =* 58)	3.07 (0.43)	3.08 (0.43)	0.01 (0.12)	0.96***	-0.2–0.26	3.11
80	Leg Press	6	Single Repetition TUT (*n =* 48)	5.68 (0.54)	5.66 (0.54)	0.02 (0.13)	0.97***	-0.26–0.25	1.83
80	Leg Press	6	Total TUT (*n =* 18)	17.57 (1.17)	16.72 (1.01)	0.86 (0.81)	0.73***	-1.97–0.37	5.2
80	Leg Press	8	Concentric TUT (*n =* 39)	3.27 (0.62)	3.24 (0.61)	0.04 (0.17)	0.96***	-0.28–0.25	4.85
80	Leg Press	8	Eccentric TUT (*n =* 45)	4.02 (0.88)	4.05 (0.91)	0.04 (0.14)	0.99***	-0.21–0.28	4.85
80	Leg Press	8	Single Repetition TUT (*n =* 46)	7.5 (0.97)	7.47 (0.96)	0.03 (0.13)	0.99***	-0.22–0.27	1.49
80	Leg Press	8	Total TUT (*n =* 18)	23.07 (2.3)	22.33 (2.2)	0.74 (0.84)	0.93***	-1.98–0.2	3.35

Abbreviations: TUT: time-under-tension

**** Denotes: *p* < 0.0001

In addition, the single contraction-specific phases were contrasted using a Mann Withney U test between metronome and actual repetition duration for both exercise machines (Table 3).

**Table 3:** Comparison of repetition velocities between metronome and participants.

Intensity [% 1-RM]	Machine	Metronome repetition velocity [s]	Actual repetition velocity [s] Median (SD)	U	*p value*
30	Leg Extension	1	1.58 (0.89)	616	0.000***
30	Leg Extension	2	2.12 (1.04)	435	0.000***
30	Leg Extension	6	5.93 (1.24)	252	0.000***
30	Leg Extension	8	7.98 (2.02)	430	0.000***
30	Leg Press	1	1.5 (0.63)	252	0.000***
30	Leg Press	2	2.1 (0.69)	82	0.000***
30	Leg Press	6	5.84 (1.35)	340	0.000***
30	Leg Press	8	7.82 (2.58)	910	0.000***
80	Leg Extension	1	1.72 (0.56)	340	0.000***
80	Leg Extension	2	2.05 (0.7)	82	0.000***
80	Leg Extension	6	5.84 (0.88)	82	0.000***
80	Leg Extension	8	7.69 (1.02)	82	0.000***
80	Leg Press	1	1.46 (0.57)	166	0.000***
80	Leg Press	2	2 (0.61)	0	0.000***
80	Leg Press	6	5.68 (1.51)	522	0.000***
80	Leg Press	8	7.56 (2.19)	616	0.000***

*n* = 27 participants

**** Denotes: *p* < 0.001

## Discussion

In this study, we show that neither variations of intensity nor velocity disrupted the algorithmic extraction of smartphone accelerometer-derived mechano-biological descriptors of resistance exercise as long as a dynamic movement is detected. As such, the temporal distribution of contraction-specific phases and total TUT can be extracted reliably and validly using smartphone accelerometer-derived data while manipulating movement velocity and/or resistance exercise intensity of a dynamic resistance exercise setting. Evidence for this finding is that even though intensity and velocity were manipulated the single repetition detection error is 1.9% when compared to the video recordings, which represented the gold standard. The mean temporal error of single repetitions, when compared to the video recordings, is 0.39%.

Low loads, *e*.*g*. 30% of 1-RM, require less force, allowing faster movement speeds. In contrast, higher loads, *e*.*g*. 80% of 1-RM are associated with higher force. Thus, movement speeds decrease following the force-velocity relationship of Hill [24]. Therefore, the acceleration decreases with increasing force production. The proposed algorithm of Viecelli *et al*. works as follows: First, the length of the three-dimensional acceleration vector is calculated and outliers are removed by a Hampel filter followed by a linear interpolation. The outliers are caused by using high-frequency (*ca*. 400 Hz) accelerometer measurements. Afterwards the gravitational offset gets corrected. To obtain the velocity, the preprocessed acceleration time-series is subjected to an integration and a polynomial fit followed by a moving average smoothing of the time-series. Although the algebraic double-integration of acceleration yields the displacement, a constant little offset error in the measured acceleration produces a quadratic baseline error on displacement calculations [25]. Therefore, the velocity domain offered an acceptable trade-off between noise and drift. Afterwards, the peaks, which are proxies for the number of repetitions while the zero-point crossings in the velocity domain reflect the reversal points (end of concentric or eccentric phase contractions) get detected. Using the time between the zero-point crossings points, the single contraction (concentric or eccentric) phase specific TUT is calculated.

As such, reversal points are the *Achilles* heel of the calculation. During dynamic exercise, time-spent at reversal points is typically short. Any acceleration-dependent algorithmic data extraction approach will fail if phases with constant and/or no velocities are introduced as acceleration becomes zero. This in turn, will lead to an algorithmic distinction problem where contraction-specific phases cannot be assigned unequivocally.

As we anticipated that intensity would not threaten the algorithmic extraction and repetition velocities ranging from 3.15–4.43 s were reliably and validly extracted by the same algorithm by Viecelli *et al*. [17], velocities of 1 s, 2 s, 6 s and 8 s per repetition were chosen. Participants tried to follow the metronome as closely as possible. We used a metronome to ensure repetition velocities. Although participants were familiarized with all the velocities, comparing the actual repetition velocities with the metronome revealed significant differences as seen in Table 3. However, as explained above, reversal points are examined and therefore the time spent in between reversal points (*e*.*g*. half-repetition velocity) does not impact the temporal extraction of the contraction-specific phases.

Hence, smartphone accelerometer-derived extraction of mechano-biological descriptors of resistance exercise works in non-static environments and are not intensity nor velocity dependent.

### Practical relevance of the results

Smartphones are ubiquitously available and equipped with a plethora of sensors that could contribute to solving health problems. As such, the automatic extraction of scientific resistance exercise mechano-biological descriptors using private smartphones could be used to systematically collect important, otherwise missing, information of clinical relevance. This information could, if available to health care professionals, be used to monitor, adjust or complement training and/or rehabilitation interventions. Additionally, such an accelerometer-derived algorithmic approach could help to standardize resistance exercise reporting.

### Limitations

Two Nexus 6P smartphones with built-in 3-axis accelerometer BMI160 (Robert Bosch GmbH, Stuttgart, Germany) were used in this study. Note that the operating system, Android (Open Handset Alliance, Maintain View, USA), is a non-real time operating system. Therefore, accelerometer-measured data values can be delayed, resulting in incorrect timestamps, or, in other instances, dropped because the device is busy [26]. Dropping or making timestamps equidistant might also have contributed to the introduction of small random temporal errors.

Because smartphone accelerometers measure proper accelerations, contraction-specific phases of dynamic resistance exercises can validly and reliably be extracted from accelerometer data. However, temporal segments without proper acceleration cannot unequivocally be assigned to any contraction-specific phases, because they could belong to isometric contractions or dynamic, constant-velocity contractions. Therefore, in a real-world scenario, our algorithmic approach could be used, whereas for isometric contractions or constant velocity movements, caution is required.

### Future research

Using smartphone accelerometers, one could imagine that volitional muscular failure should be detectable as the force-velocity relationship postulates slower velocities with increasing force generation [24]. Therefore, towards total exhaustion, velocity will decrease and should be significantly different when compared the *e*.*g*. the first repetition.

## References

[1] ACSM. Progression models in resistance training for healthy adults. Med Sci Sports Exerc. 2009;41: 687–708. doi: 10.1249/MSS.0b013e3181915670 19204579

[2] Dudley GA, Tesch PA, Miller BJ, Buchanan P. Importance of eccentric actions in performance adaptations to resistance training. Aviat Sp Environ Med. 1991; 1859341

[3] Franchi M V., Reeves ND, Narici M V. Skeletal muscle remodeling in response to eccentric vs. concentric loading: Morphological, molecular, and metabolic adaptations. Front Physiol. Frontiers; 2017;8: 447. doi: 10.3389/fphys.2017.00447 28725197 PMC5495834

[4] Coburn JW, Housh TJ, Malek MH, Weir JP, Cramer JT, Beck TW, et al. Neuromuscular responses to three days of velocity-specific isokinetic training. J Strength Cond Res. United States; 2006;20: 892–898. doi: 10.1519/R-18745.1 17194247

[5] Coyle EF, Feiring DC, Rotkis TC, Cote RW, Roby FB, Lee W, et al. Specificity of power improvements through slow and fast isokinetic training. J Appl Physiol Respir Environ Exerc Physiol. 1981;51: 1437–1442. doi: 10.1152/jappl.1981.51.6.1437 7319877

[6] Burd NA, Andrews RJ, West DWD, Little JP, Cochran AJR, Hector AJ, et al. Muscle time under tension during resistance exercise stimulates differential muscle protein sub-fractional synthetic responses in men. J Physiol. John Wiley & Sons, Ltd; 2012;590: 351–362. doi: 10.1113/jphysiol.2011.221200 22106173 PMC3285070

[7] Knapik JJ, Mawdsley RH, Ramos MU. Angular specificity and test mode specificity of isometric and isokinetic strength training. J Orthop Sports Phys Ther. 1983;5: 58–65. doi: 10.2519/jospt.1983.5.2.58 18806429

[8] Kraemer WJ, Nindl BC, Ratamess NA, Gotshalk LA, Volek JS, Fleck SJ, et al. Changes in Muscle Hypertrophy in Women with Periodized Resistance Training. Med Sci Sports Exerc. 2004;36: 697–708. doi: 10.1249/01.mss.0000122734.25411.cf 15064598

[9] Tesch PA, Thorsson A, Essen-Gustavsson B. Enzyme activities of FT and ST muscle fibers in heavy-resistance trained athletes. J Appl Physiol. 1989;67: 83–98. doi: 10.1152/jappl.1989.67.1.83 2547751

[10] Rhea MR, Alvar BA, Burkett LN, Ball SD. A meta-analysis to determine the dose response for strength development. Med Sci Sports Exerc. 2003;35: 456–464. doi: 10.1249/01.MSS.0000053727.63505.D4 12618576

[11] Tanimoto M, Ishii N. Effects of low-intensity resistance exercise with slow movement and tonic force generation on muscular function in young men. J Appl Physiol. 2006;100: 1150–1157. doi: 10.1152/japplphysiol.00741.2005 16339347

[12] Watanabe Y, Tanimoto M, Ohgane A, Sanada K, Miyachi M, Ishii N. Increased muscle size and strength from slow-movement, low-intensity resistance exercise and tonic force generation. J Aging Phys Act. 2013;21: 71–84. doi: 10.1123/japa.21.1.71 22832536

[13] Watanabe Y, Madarame H, Ogasawara R, Nakazato K, Ishii N. Effect of very low-intensity resistance training with slow movement on muscle size and strength in healthy older adults. Clin Physiol Funct Imaging. 2014;34: 463–470. doi: 10.1111/cpf.12117 24304680

[14] Pereira PEA, Motoyama YL, Esteves GJ, Quinelato WC, Botter L, Tanaka KH, et al. Resistance training with slow speed of movement is better for hypertrophy and muscle strength gains than fast speed of movement. Int J Appl Exerc Physiol. 2016;5: 2322–3537.

[15] Hackett DA, Davies TB, Orr R, Kuang K, Halaki M. Effect of movement velocity during resistance training on muscle-specific hypertrophy: A systematic review. Eur J Sport Sci. 2018;18: 473–482. doi: 10.1080/17461391.2018.1434563 29431597

[16] Toigo M, Boutellier U. New fundamental resistance exercise determinants of molecular and cellular muscle adaptations. European Journal of Applied Physiology. 2006. pp. 643–663. doi: 10.1007/s00421-006-0238-1 16845551

[17] Viecelli C, Graf D, Aguayo D, Hafen E, Füchslin RM. Using smartphone accelerometer data to obtain scientific mechanical-biological descriptors of resistance exercise training. Dimitriadis SI, editor. PLoS One. Public Library of Science; 2020;15: e0235156. doi: 10.1371/journal.pone.0235156 32667945 PMC7363108

[18] Mayhew JL, Ball TE, Arnold MD, Bowen JC. Relative muscular endurance performance as a predictor of bench press strength in college men and women. J Strength Cond Res. 1992;6: 200–206. doi: 10.1519/00124278-199211000-00002

[19] Keep H, Luu L, Berson A, Garland SJ. Validity of the handheld dynamometer compared with an isokinetic dynamometer in measuring peak hip extension strength. Physiother Canada. 2016;68: 15–22. doi: 10.3138/ptc.2014-62 27504043 PMC4961312

[20] Hampel FR. The influence curve and its role in robust estimation. J Am Stat Assoc. 1974;69: 383–393. doi: 10.1080/01621459.1974.10482962

[21] Martin Bland J, Altman DG. Statistical Methods for Assessing Agreement Between Two Methods of Clinical Measurement. Lancet. 1986;327: 307–310. doi: 10.1016/S0140-6736(86)90837-8 2868172

[22] Altman DG, Bland JM. Measuring agreement in method comparison studies. Stat Methods Med Res. 1999;8: 135–160. doi: 10.1177/096228029900800204 10501650

[23] Sachs L, Hedderich J. Angewandte Statistik. In: Angewandte Statistik [Internet]. 2006 [cited 5 Mar 2020] p. 721. doi: 10.1007/978-3-540-32161-3

[24] Hill A V. The Heat of Shortening and the Dynamic Constants of Muscle. Proc R Soc B Biol Sci. 1938;126: 136–195. doi: 10.1098/rspb.1938.0050

[25] Feltrin G, Gsell D, Weber F. Berechnung von Verschiebungen mittels Zeit-Integration gemessener Beschleunigungen: Eine kleine Untersuchung. 2004.

[26] Milette G, Stroud A. Professional Android Sensor Programming [Internet]. John Wiley & Sons; 2012. doi: 978-1-118-18348-9

